# Characterization of a new natural novel lignocellulose fiber resource from the stem of *Cyperus platystylis* R.Br.

**DOI:** 10.1038/s41598-023-35888-w

**Published:** 2023-06-15

**Authors:** Anup Kumar Bhunia, Dheeman Mondal, Sanjukta Mondal Parui, Amal Kumar Mondal

**Affiliations:** 1 Plant Taxonomy, Biosystematics and Molecular Taxonomy Laboratory, UGC-DRS-SAP-II and DBT-BOOST WB Supported Department, Department of Botany and Forestry, Vidyasagar University, Midnapore, 721102 West Bengal India; 2 Biochemistry Laboratory, Post Graduate Department of Zoology, Lady Brabourne College, P1/2, Suhrawardy Avenue, Kolkata, 700017 West Bengal India

**Keywords:** Biochemistry, Biophysics, Plant sciences, Environmental sciences

## Abstract

This study deals with the characterization of a natural fiber which is extracted from the stem of an unexplored plant of *Cyperus platystylis* R.Br. (CPS) with an aim to establish it as a potent alternative fiber for the plant fiber-based industries. CPS fiber has been investigated for its physical, chemical, thermal, mechanical, and morphological characteristics. The presence of different functional groups in CPS fiber i.e., cellulose, hemicellulose, and lignin which was ensured by Fourier Transformed Infrared (FTIR) Spectrophotometer analysis. X-ray diffraction and chemical constituent analysis revealed high cellulose content and crystallinity i.e., 66.1% and 41.12% respectively, which is comparatively moderate in the case of CPS fiber. Scherrer's equation has been used to determine crystallite size i.e., 2.28 nm. The mean length and diameter of the CPS fiber were 382.0 and 23.36 μm, respectively. The maximum tensile strength was obtained at 657 ± 58.8 MPa for 50 mm fiber and young’s modulus 88.76 ± 30.42 MPa for 50 mm fiber. The required energy to break has been recorded at 346.16 J. Thermal analysis revealed that CPS fibers have thermal stability up to 279 °C. The unique *Cyperus platystylis* stem fibers could therefore be a suitable reinforcement material for the bio-composites used in semi-structural applications since they have higher functional qualities.

## Introduction

The last century has seen a revolution in the world of polymers and plastics with the introduction of many plastic and synthetic polymers. These have become a part of our everyday lives, and these synthetic fibers have gained prominence due to their high specific strength, low weight, and durability. In addition to the financial and environmental advantages, natural fibers also have a number of other advantages over synthetic alternatives, including low density, improved thermal insulation capabilities (for use in, for instance, automobiles and buildings), equivalent mechanical properties, reduced wear, etc.^1–7^. Synthetic and plastic polymers are currently used as raw materials in various industries and are in high demand in various industrial sectors. They are also frequently utilized in various applications, including as in the packaging, textile, aerospace, and household and sports appliance industries^8–16^. But these synthetic polymers have some major drawbacks that include high cost, high energy consumption, and the most remarkable is non-biodegradable. The use of natural fibers as reinforcing agents in polymer composites has increased recently in response to worries about environmental pollution and degradation brought on by petroleum-based synthetic materials^17^. Thus, in the last few decades, natural resources and crop-leftover have been considered as alternatives to synthetic fibers and potential uses for natural fibers in automobiles include doors, bumpers, and brakes, etc.^18–24^. Natural fibers have the advantage over synthetic fibers and are the most promising and reliable resource for industry because of their green properties viz. lower cost and weight, greater tensile strength, stiffness, thermal stability, thermal insulation, resistance to water, non-toxic nature, bio-renewable and bio-degradable too^7,25^. Due to environment-related awareness, researchers, scientists, and industrialists are developing sustainable materials^26^. Natural fiber-producing plants can control the emission of toxic chemicals and non-biodegradable waste generation during the manufacturing process, unlike the synthetic fiber-based manufacturing plant. The adaptation of natural fibers can open up a new window in agriculture or agriculture-based industries for raw materials. Generally, natural fibers extracted from different regions of plants like, leaf, stem, roots, fruits, etc. are used in a cottage or the textile industry with a combination of resin or other things that do not irritate when they come in contact with the skin. Cellulose, hemicellulose, lignin, and wax are the major components of natural fiber. These substances do not adversely affect Human beings. Several researchers around the world have been working on traditional fibers like cotton, jute, rice husk, kenaf, sugarcane, coconut fiber, pineapple fiber, etc.^27,28^.

In this work, natural fiber was extracted from the stem of the plant *Cyperus platystylis,* which grows extensively in the wetland areas of the Indian subcontinent^29^. *Cyperus platystylis* belongs to the family Cyperaceae. It belongs to the most diverse family of flowering plants, with about 5000 species. *Cyperus platystylis* is a sedge species typically found in South-east Asia and Australia. Robert Brown first formally described this species in 1810^30^. The CPS fibers are eco-friendly, inexpensive, and easily available as a result renewable. Hence technical characterization was crucial for this fiber. This study explored and identified alternative new fiber-yielding species and novel uses of these fibers through R & D as raw materials in plant fiber-based industries. This will help decrease the pressure on the handful number of species used for fiber, leading to the exhaustion of this species forest resources, which in turn causes of the significant loss of biodiversity. The morphological characteristic features and the physical, thermal, chemical and mechanical features of this newly identified natural fiber resource have been characterized.

## Materials and methods

### Extraction of CPS fibers

**Figure 1:**
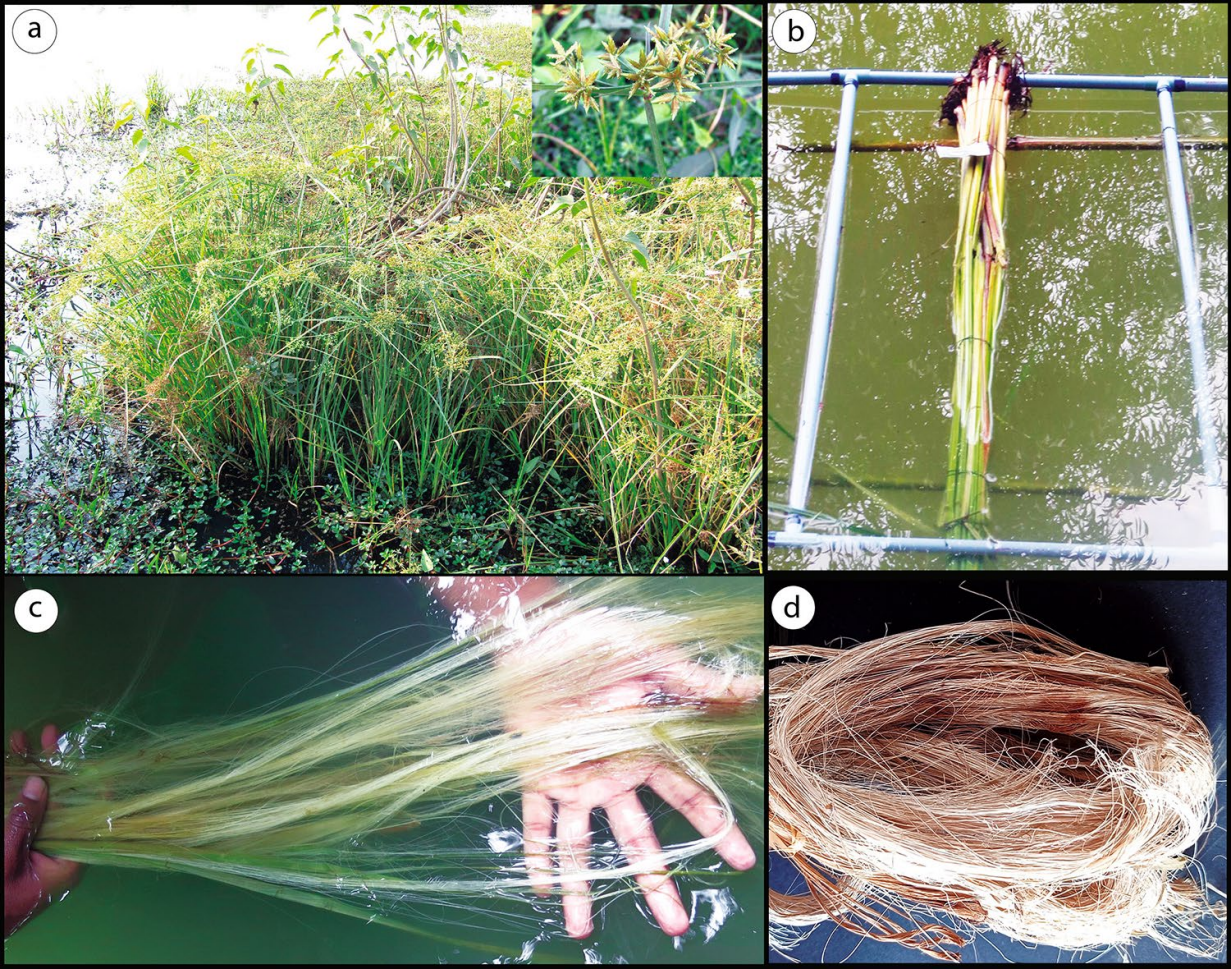
Extraction of fiber from *Cyperus platystylis* stem (**a**) *Cyperus platystylis* in its natural habitat (**b**) *Cyperus platystylis* stem immersed in water for fiber extraction (**c**) Fiber extraction in immersed water (**d**) Fibers after extraction.

**Figure 2:**
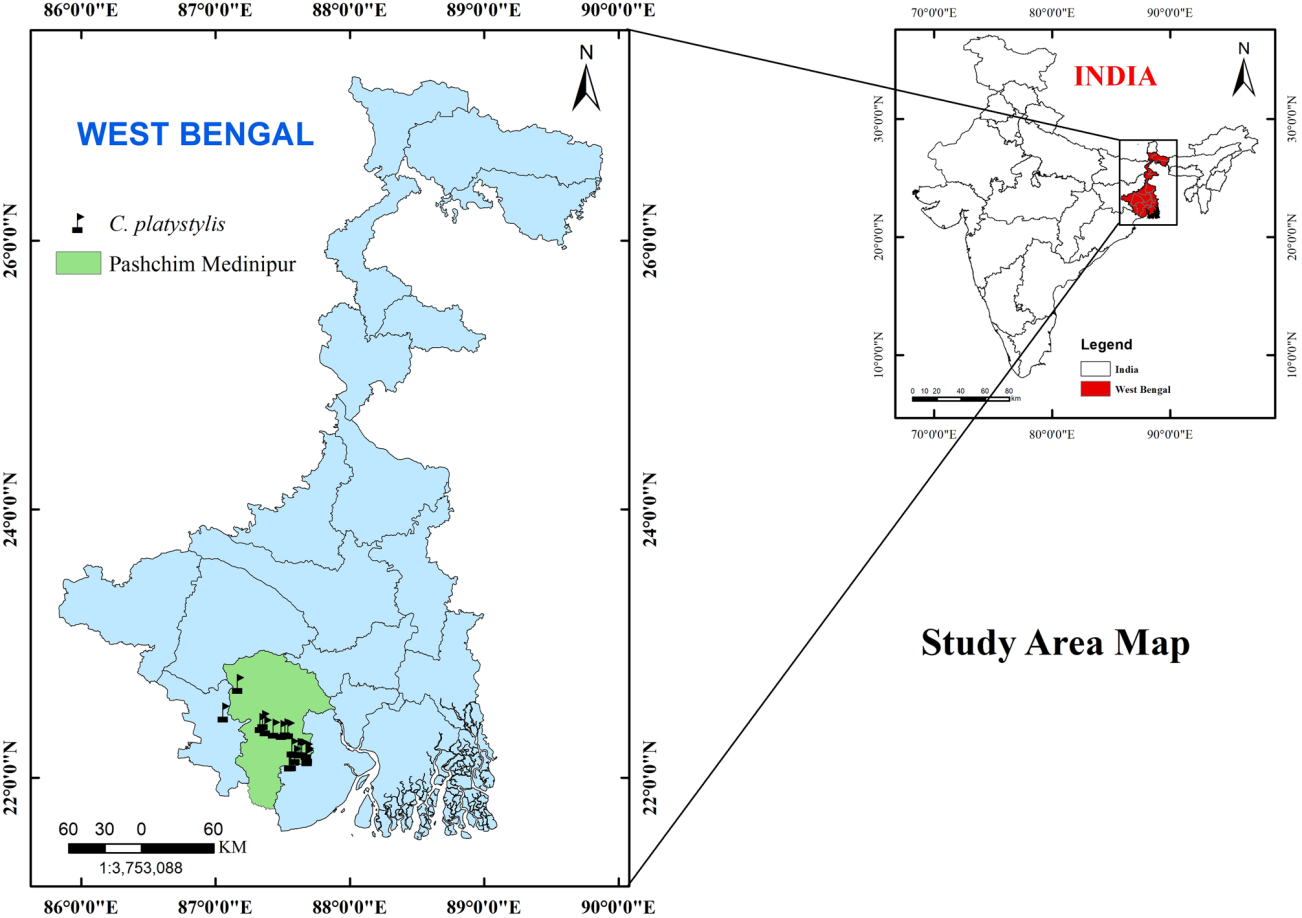
Study area map with GIS information prepared through Arc GIS Pro ver.2021.

*Cyperus platystylis* plants were collected from the Paschim Medinipur district of West Bengal, India. GPS coordinates of the collected sample are E 87° 22′ 51.8628′′, N 22° 24.4823′′. The plant sample was identified and assigned the accession number of VU/Anup/002 by Vinay Ranjan, Scientist D, Central National Herbarium, Botanical Survey of India (BSI). Voucher specimen is deposited into the repository of Vidyasagar University Herbarium section (Fig. 1). All procedures were conducted in accordance with the guidelines. As an agricultural weed the plant is grown on the field. The plant collection is done with prior permission from competent authority. A study map has also been prepared through Geographic Information System to earmark the proper location of the sample for future researchers (Fig. 2). The plants were collected from the field and the stem excised from the plants. To obtain fiber from the selected specimen retting by water^31^ was performed for this experiment (Fig. 1a–d). The stem parts were left fully merged in normal water for 20–25 days for in air microbial degradation, allowing the cellular tissues and pectin surrounding bast-fiber bundles to rot away, by doing so, the fiber could be extracted out from the stem more easily than if it had been separated manually (using metal brush). The separated fibers were thoroughly cleaned to discard the debris and then thoroughly washed with water (Double distilled) repeatedly several times. The fibers were dried under indirect sunlight for two to three days and then kept under ambient conditions for one week for their characterization.

### Physical characterization

**Figure 3:**
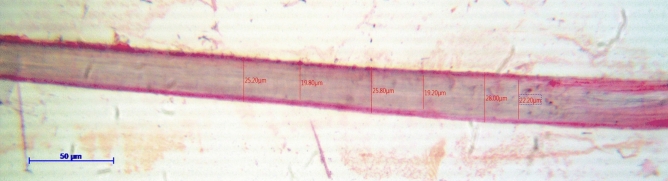
Optical Microscopic photograph of *Cyperus platystylis* stem fiber.

The CPS fiber was countervailed under standard conditions i.e., 27 °C temperature and 65% relative humidity for 24 h. To measure the fiber diameter, the fiber bundles were randomly chosen and crushed using a mortar and pestle. Crushed fibers (Fig. 3) were taken randomly onto slides for optical microscopy and the process of the test was repeated three times. The fiber length, width, lumen diameter, and cell wall thickness (Fig. 3) were calculated under an optical microscope (Leica DM1000). The Slenderness Ratio^32,33^, Flexibility Coefficient^32,33^, and Runkel Ratio^32,33^ were obtained from the physical characteristic data.

1 $$\mathrm{Slenderness\ Ratio }\left(\mathrm{SR}\right)=\frac{{l}_{f}}{{D}_{f}}$$

where* l*_*f*_ represents fiber length and *D*_*f*_ represents fiber diameter (all units in µm).

2 $$\mathrm{Flexibility\ Coeffiient}=\frac{{LD}_{f}}{{D}_{f}}\times 100$$

The fiber lumen diameter is represented as *LD*_*f*_ and *D*_*f*_ represents fiber diameter (all units in µm).

3 $$\mathrm{Runkel\ Ration }\left(\mathrm{RR}\right)=\frac{{CW}_{t}}{{LD}_{f}}\times 2$$

Here, *CWt* represents cell-wall thickness and *LD*_*f*_ fiber lumen diameter (all units in µm).

### Chemical characterization

Chemical characterization of the extracted fibers was performed to determine the cellulose, hemi-cellulose, and lignin content following conventional methods^34,35^. 1gm of finely powdered fiber was taken for each analysis.

4 $$Total\ Cellulose\left(\%\ on\ dry\ basis\right)=\frac{Residue}{Dry\ material\ weight}\times 100$$

5 $$Hemicellulose\ content=\frac{Neutral\ detergent\ fiber\left(NDF\right)-Acid\ detergent\ fiber(ADF)}{Total\ Initial\ Weight}\times 100$$

6 $$Lignin\left(\%\ on\ dry\ basis\right)=\frac{Residue}{Dry\ material\ weight}\times 100$$

### Spectrometric characterization (FTIR)

**Figure 4:**
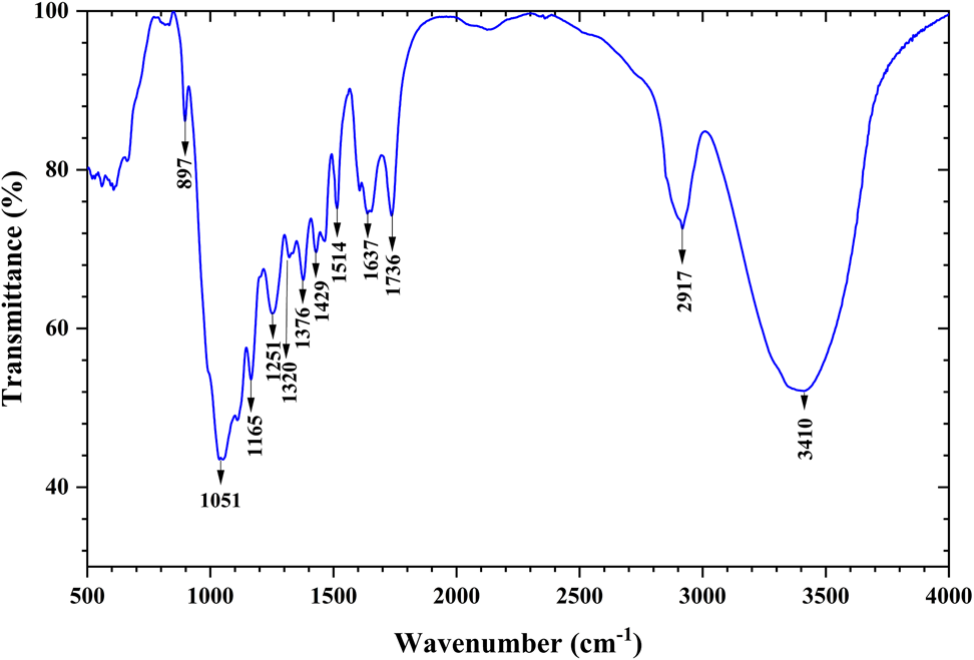
FTIR spectrogram of CPS fiber.

The functional groups present in CPS fiber have been measured using Fourier Transformed Infrared (FTIR) Spectrophotometer (Thermo fisher Nicolet Avatar Model 360 spectrometer, United States)^36,37^. For measurement, the substance was crushed into a fine powder and carefully mixed using potassium bromide (KBr). The pellets were prepared using 1 mg fiber powder mixed with 100 mg Potassium Bromide (KBr). FTIR spectra were recorded in the wave- number region from 500–4000 cm^−1^and resolution of 2 cm^-1^with a scan-rate of 32 scans per minute at room temperature with 65% relative humidity (Fig. 4).

### X-ray diffraction (XRD)

**Figure 5:**
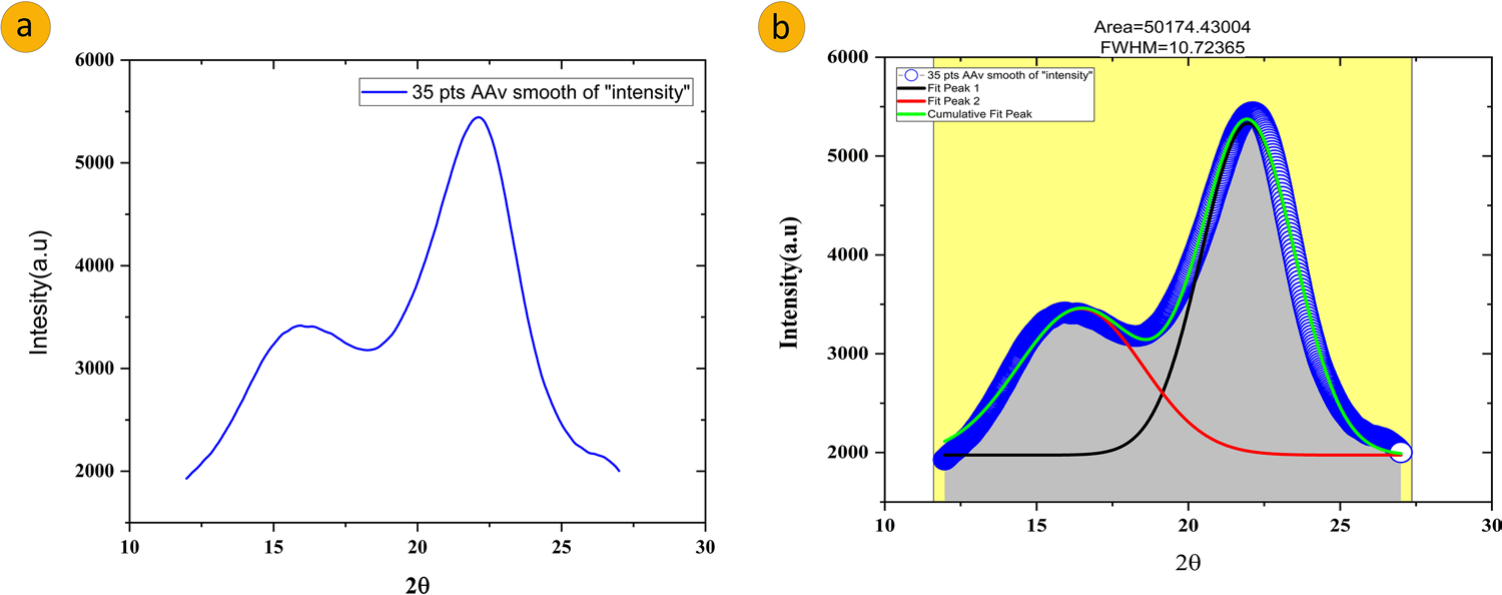
X-ray diffraction pattern of CPS fiber (**a**) Showing XRD pattern 35pts adjacent-average smoothing of intensity in Origin Pro 2021 software (**b**) Two significant peaks are fitted in Gaussian for the analysis and Cumulative data is also represented.

The X-Ray diffraction (XRD) method was used to assess the Crystallinity index (CI) of the CPS fiber. The purpose was to assess the Crystalline Index (CI) and Crystal Size of separated CPS fiber. This was done using X’Pert Pro-PAN analytical software (Bruker-ASX, Germany) with the aid of CuKα radiation generated at a voltage of 30 kV and a current of 10 mA. Firstly, the sample was crushed with mortar and pestle to fine powder for this study. The sample was evaluated at a temperature (temp.) of 25 °C in 2θ ranging from 10° to 80° using step size of 0.02°. The wavelength of X-Ray radiation is 0.1542 nm in the reflection mode. All have the maximum intensity of 5500 counts. A peak fitting program (Origin Software, 2021) was used to smooth the peaks up to 35pts by signal processing by adjacent-averaging (Fig. 5a), assuming a Gaussian function for each peak and a broad peak at around 16.44° (Fig. 5b). In all cases, F number was > 10,000, which corresponds to a R^2^ value > 0.99855.

The CI was determined with the aid of the below-mentioned formula^19,38^.

7 $$Crystallinity\ index=\frac{{I}_{C}}{({I}_{C}+{I}_{a})}\times 100$$

where *I*_*c*_ is the intensity of crystalline component and *I*_*a*_ is the intensity of amorphous component.

8 $$Crystal\ Size=\frac{k\uplambda }{\beta cos\theta }$$

The Crystal Size (CS) of the CPS fiber had been calculated with the aid of Scherrer’s formula given Eq. 8^39^, by the wavelength of the incident X-Ray radiation (λ = 0.1542 nm), Scherrer’s correction factor (k = 0.94), peak’s full width half maximum (β) and Bragg angle (θ).

### Mechanical properties

Tensile properties were ascertained with a Tinius Olsen H50KS Universal-Testing Machine at 0.5 mm/minute crosshead speed while maintaining a gauge length of 50 mm. Ten CPS fibers were tested to obtain statistically significant data. All experiments were conducted at an ambient temperature of 25 °C with 65% relative humidity^40–42^. Tensile strength, elongation of break, and young’s modulus were calculated to ascertain the mechanical strength or durability of the selected fiber. The following equations were used to compute the tensile-strength and young's modulus. Breaking Load, Cross-sectional area, Stress, and Strain were used to compute the test data.

9 $$Tensile\ strength\left(\frac{N}{{mm}^{2}}\right)=\frac{Breaking\ Load(N)}{Cross\ Section\ Area}$$

10 $$Youn{g}^{^{\prime}}s\ modulus=\frac{Stress}{Strain}$$

### Thermal analysis (thermogravimetric analysis)

The Thermogravimetric analyser (Perkin Elmer, Model-Pyris Diamond TG/DTA, United States) was employed to analyse the thermal durability of the selected fibers. The thermograms of CPS fibers were taken in a nitrogen environment at a heating rate of 10 °C/min and temperatures ranging from 50–600 °C^43^.

The kinetic energy (E_a_) of the CPS fiber was computed using Broido’s Eq. (11)^44^. It is the least energy required to break down fibers that is represented as activation energy. Broido’s equation is as follows-

11 $$\mathrm{ln}\left[\mathrm{ln}\left(\frac{1}{y}\right)\right]=\left(\frac{E}{R}\right)[\left(\frac{1}{T}\right)+K]$$

where, y indicates the normalized weight (W_t_/W_0_), Wt is the weight of the plant sample at any time t, W_0_ is the sample's baseline weight, T is the kelvin temperature, and R is the Universal Gas Constant (8.32 kJ/mol-k).

Two exothermic spectrums have been recorded in the derivative thermogravimetry (DTG) data of CPS fiber. Based on the derived data, it is possible to determine how much mass is lost in each phase of degradation.

### Ethics approval and consent to participate

Authors declare that the submitted work should be original and should not have been published elsewhere in any form or language. The manuscript data is a single study not been spelled up into several parts to increase the quantity of submissions and submitted to various journals or to one journal over time. Results presented clearly, honestly, and without fabrication, falsification, or inappropriate data manipulation (including image-based manipulation).

## Morphological studies

### Scanning electron microscopy (SEM)

A Scanning Electron Microscopy (ZEISS Supra-40) was used for examining the surface topology of the selected fiber samples. SEM was used for studying the fiber-matrix adhesion. The SEM studies were performed by scanning the fiber sample with a high-energy electron beam at an accelerating voltage of 5 kV in a secondary electron imaging mode. Eventually, the sample surface shown at differential magnifications was taken as the resulting image. For proper conductivity of the CPS fiber, this sample was coated with gold in a vacuum chamber before the analysis.

### Energy-dispersive X-ray spectroscopy (EDX)

EDX is a very common and useful method for proper identification of elements viz. Oxygen, Carbon, Nitrogen, etc. The presence of various elements on CPS fiber was tested using EDX analysis, which is fully equipped with SEM.

### Surface roughness analysis

A non-contact 3D profiler (Bruker Nano GmbH, Germany) was exercised to measure the Surface Roughness (SR) of the selected fibers. At least five samples were exercised to get the average result. The outcomes of the test results have been recorded and average values have been observed. A minimal 50 mm fiber-length was taken for measurement. The measurement continued along the length of the fiber.

## Results and discussion

### Physical characteristics

The CPS fiber length varies from 220 to 593.09 μm and the mean-length of a single fiber is 382.0 μm. The CPS fiber diameter ranges from 22.20 to 28.00 μm and the mean-diameter of the CPS fiber is 23.36 μm. Lumen diameter and cell-wall thickness of CPS fiber are 10.77 μm and 3.90 μm respectively. The Slenderness ratio (SR) of the CPS fiber is 32.52, and the flexibility-coefficient and Runkel ratio (RR) are 57.96 and 0.72 respectively. It was extremely difficult to ascertain the exact fiber length as the CPS fibers are clumped together as a bundle from which the extraction of individual fiber was quite tough. Environmental conditions and other factors also affect the growth of natural resource-based fibers. Hence, uniform length and width are impossible for a single fiber^45^. The fiber-length and diameter of *Cyperus platystylis* were found to be rather comparable to CPF_S_*,* hemp, flax, and jute fibers, as represented in Table 1.

**Table 1:** Comparative data of physical and chemical features of CPS fiber with other natural resource-based fibers.

Fiber	Physical Properties		Chemical Properties		
	Length (μm)	Diameter (μm)	Cellulose (%)	Hemicellulose (%)	Lignin (%)
CPS Fiber	220–593.09	22.20–28.00	66.1	20.12	13.28
CPFs^45^	602.06	16.20	68.5	–	17.88
Flax fiber^69^	–	20–25	71	18.6–20.6	2.2
Hemp fiber^69^	–	28–38	68	15	10
Ramie fiber^69^	–	24	68.6–85	13–16.7	0.5–0.7
Pineapple fiber^69^	–	–	81	–	12.7
Bamboo fiber^69^	–	–	26–43	30	21–31
Abaca fiber^69^	–	–	56–63	20–25	7–9
Borassus fiber^69^	–	–	53.4	29.6	17
Napier fiber^69^	–	–	45.66	33.67	20.60
Jute^55^	–	25–200	61–71	12–20	12–13
Cotton^55^	–		82.7	5.7	–
Banana^55^	–	60–250	65	19	5
Coconut/Coir^55^	–	100–450	4.2	12.1	32.8
CTF fiber^55^	–	45–548	5.1	21.78	17.6

### Chemical characterization

The main components of this lignocellulosic fiber were Cellulose, Hemi-cellulose, and lignin, and the amounts in the CPS fiber were 66.10%, 20.12%, and 13.28% respectively. The tensile behaviour of the CPS fiber increases due to the existence of cellulose, which is regarded as the principal component of natural resource-based fibers. Table 1 gives a comparative account of the chemical makeup of CPS fiber and other natural resource-based fibers. The cellulose content is more or less similar to CPFs, Hemp, Ramie, Jute, and Banana fiber. Hemicellulose amount in the CPS fiber is more as comparable to other natural resource-based fibers. The lignin content influenced the structure, morphology, and rigidity of fibers, and the content was remarkable in the case of CPS fiber, however less than Napier, Bamboo, and CPFs fiber.

### Spectroscopic characterization (FTIR)

Figure 4 depicts the FTIR study of CPS fiber in 500–4000 cm^−1^ wave number band, performed to ascertain the appearance of chemical contents in the crude fiber. The absorption spectra depict various functional groups in ligno-cellulosic fiber i.e., cellulose, hemi-cellulose, and lignin. A strong absorption peak was investigated in the range of 3400-3423 cm^−1^ (Fig. 4), which represents the O–H stretching in the carboxylic group of cellulose^46^. The strong absorption peak at 2917 cm^−1^ corresponds to the C–H stretching of cellulose^47,48^. This indicates the existence of cellulose in CPS fiber. The spectra at 1736 cm^−1^ and 1637 cm^−1^ were assigned to C=O stretching vibrations for the acetyl groups in lignin and hemicellulose^49–51^. The 1514 cm^−1^ and 1429 cm^−1^peaks indicate the C=C aromatic skeletal vibration of lignin^52^. The absorbance spectrum around 1320–897 cm^−1^ indicates β-glycoside linkage of cellulose^42,53,54^.

### X-ray diffraction (XRD)

The XRD pattern of CPS fiber is represented in Fig. 5a,b. The diffractogram showed two reflections, corresponding to 2θ values of around 16.44° and 21.96° (Fig. 5b)*,* respectively. Among all the peaks, the lesser angle reflection (16.44°, FWHM value is 4.85°) was of low intensity, denoting of amorphous material such as cellulose, hemicellulose and lignin^26,55^ and the other reflection (21.96°, FWHM value is 3.63°) had comparatively higher intensity and represented by crystalline material can be attributed to cellulose IV in cellulosic fiber. The lower intensity peak is denoted by 18.36° for Crystallinity index calculation. Gaussian functions^56^ are commonly used for the deconvolution of XRD spectra. CI is calculated from the ratio of the area of all crystalline peaks to the total area. An important assumption for this analysis is that increased amorphous contribution is the main contributor to peak broadening. Cellulose peaks are very broad and not well resolved, with overlapping peaks. It is generally accepted in the cellulose community that peak broadening is due to the amorphous cellulose^57^. The Crystallinity Index had been calculated as 41.12%, which is comparatively lower than that of cotton (60%), jute (71%), flax fiber (80%) and Hemp (88%)^41,46,58–61^ but considerably higher than Palm-fiber (19.9%) and Coconut-fiber (19.9%). The crystallinity index of CPS fibers is greater than other natural fibers i.e., kapok and balsa fibers^62^.

The Crystallite Size of the CPS fibers calculated from Scherrer’s equation represents that the Crystal size value of CPS fibers is 2.28 nm. Cellulose peaks are very broad and not well resolved, with overlapping peaks. It is generally accepted in the cellulose community that peak broadening is due to amorphous cellulose. The crystallite size of the selected fibers is comparatively lesser than jute, and sisal, but greater than ramie, and wheat straw fiber^63,64^.

### Mechanical features

**Figure 6:**
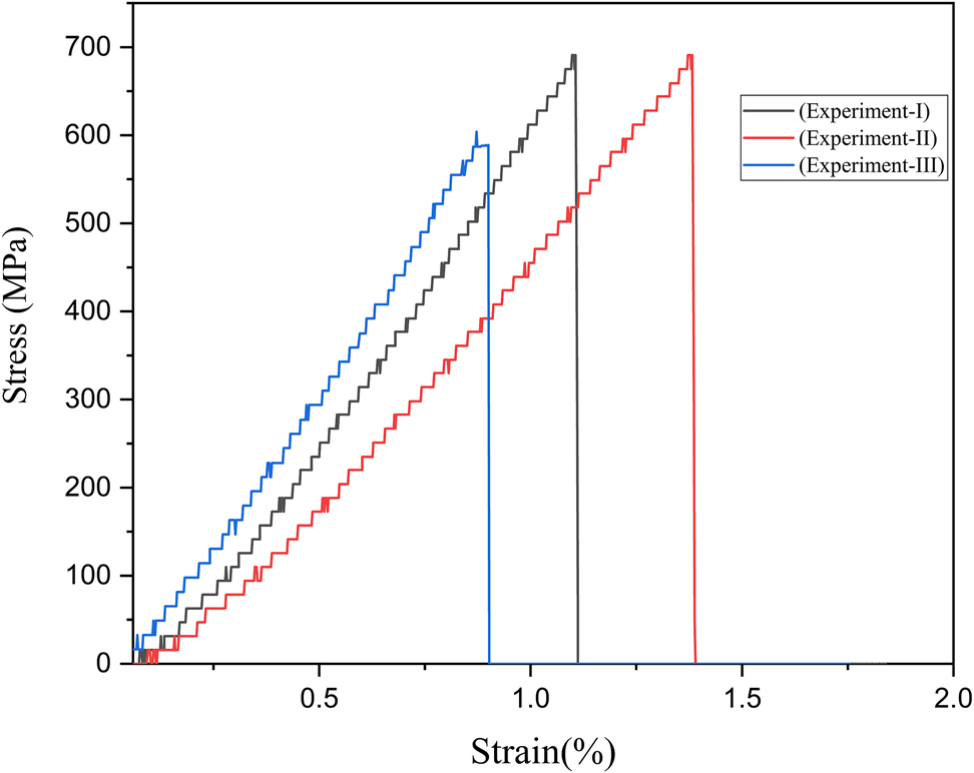
Tensile stress (%)-strain (MPa) graph of CPS fiber.

The tensile behavior of the CPS fiber was studied to examine the mechanical features of the CPS fiber under investigation and the results are in Table 2 and represented in Fig. 6. Young's modulus, energy at the break, elongation at the time of break, and strength of the CPS fiber, are the four important characteristics visible in tensile strength as represented in Table 2 and Fig. 6. The effective gauge length is 50 mm. Young's modulus is ascertaining the elasticity zone of the stress strain curve of each experiment. Young's modulus value shows only 88.76 ± 30.42 GPa. The CPS fiber exhibits the assert energy at the time of break is 346.16 J.

**Table 2:** Tensile features of CPS fiber.

The name of the fiber	Diameter (mm)	Effective gauge length (mm)	Strength (MPa)	Elongation at the break (%)	Young’s modulus (GPa)	Break energy (J)
CPS Fiber	0.052	50	657 ± 58.8	0.78 ± 0.28	88.76 ± 30.42	346.16

### Thermal analysis

**Figure 7:**
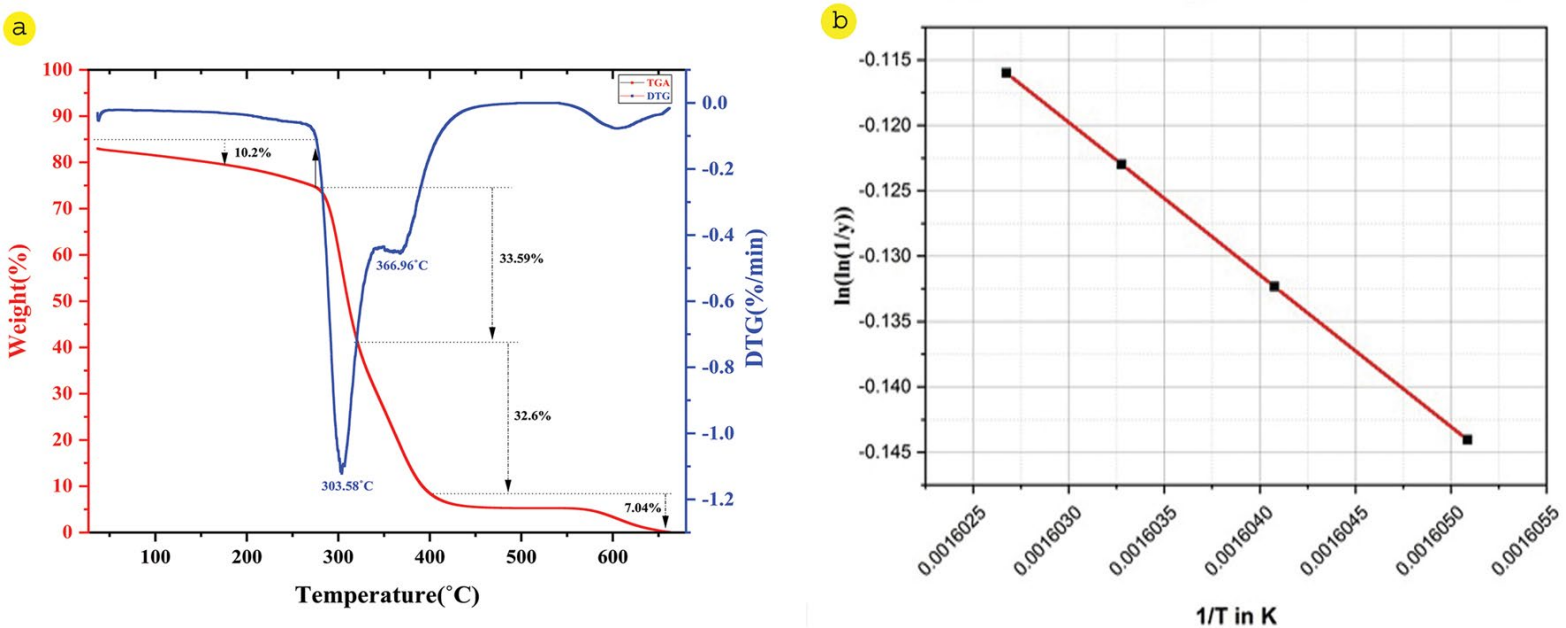
(**a**) TGA Graph, (**b**) Broido’s plot of CPS fiber.

The thermal analysis of CPS fiber was performed with the assistance of a thermogravimetric analyzer (TGA). Derivative thermo-gravimetry (DTG) and Broido’s plot are presented in Figs. 7a,b, respectively. Natural fiber under extreme heat conditions degrades in the following sequence: moisture, hemi-cellulose, cellulose and lignin, and the residuum of the constituents. Figure 7a indicates the evaporation of moisture from the CPS fiber at 36–89 °C. During this stage, the change in mass is about 10.2% of the initial mass. In the second step of break down, cellulose and lignin are degraded at 279 °C and 342 °C and overall mass loss in this stage is a maximum of around 66.19%. The final step of degradation occurred at the range of 403–660 °C where the cellulose mainly degrades, which resembles the decay of lignin and wax, which waive ash residue^65–68^. The kinetic-activation energy (E_a_) is required for preceding of the CPS fiber break down, as represented in Broido’s plot (Fig. 7b) and the requisite energy is 96.74 kJ/mol. The TGA investigation of the CPS fiber reveals that this fiber can be exercised as a more effective reinforcement in different industrial applications.

### Scanning electron microscope analysis

**Figure 8:**
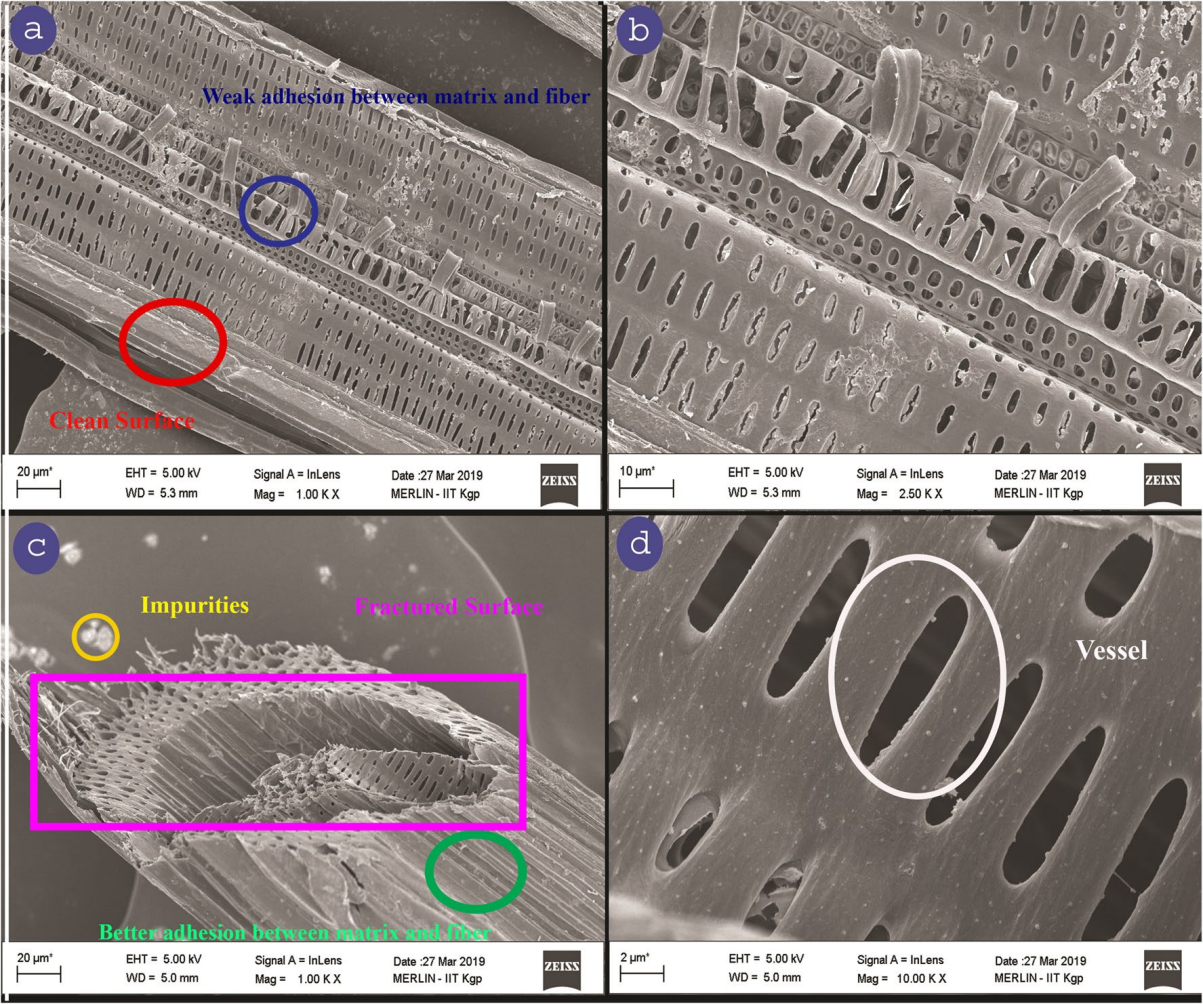
(**a**–**d**) SEM photographs of CPS fiber.

The surface topology of the CPS fiber has been done by Scanning Electron Microscope and is presented in Fig. 8a–d. The SEM photograph shows the longitudinal appearance of the CPS fiber surface under lower and higher magnifications. The rough and more uneven surface morphology of CPS fiber is observed in Fig. 8c. Vessels are clearly seen in Fig. 8a,b,d Cracks, micro-voids, and impurities emerge on the surface of CPS fibers, according to this morphology. An example of a cross-sectional SEM picture of a shattered CPS fibre following a tensile test is shown in Fig. 8c. Clarifying the shape of a cracked CPS fibre and correlating SEM micrographs with the findings of mechanical characteristics are the key goals of fracture surface analysis. The micrograms of the transverse-section of CPS fibers are represented in Fig. 8b,c from which, CPS fibers are multi-cellular. In cross-sectional appearance, the CPS fibers are mainly polygonal, with rounded corners and oval to round lumens.

### Energy dispersive X-Ray (EDX) composition analysis

**Figure 9:**
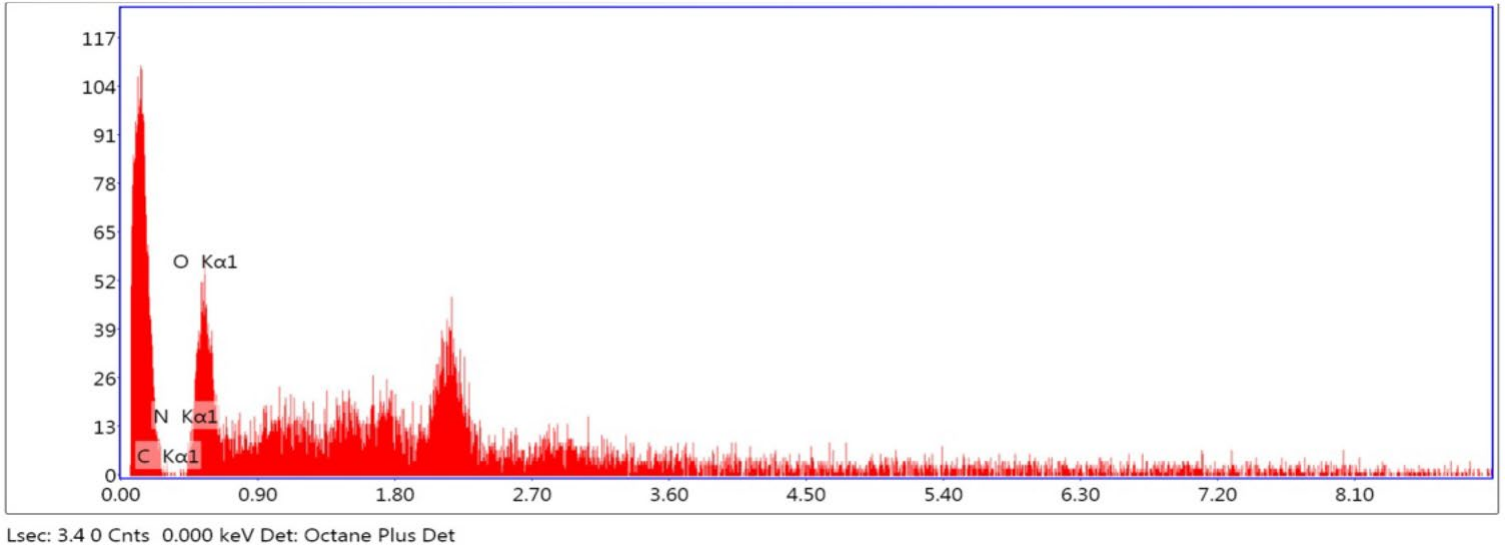
EDX result of CPS fiber.

In Fig. 9, the quantitative component exploration of the CPS fiber is depicted in respect to atomic and weight percentage. The primary elements of the CPS fiber are carbon, nitrogen, and oxygen (O), which are the principal elements of the CPS fiber surface. The atomic and weight carbon percentages are 73.75, and 67.85, nitrogen is 0.16 and 015, and oxygen is 31.99 and 26.10 respectively.

### Surface roughness

**Figure 10:**
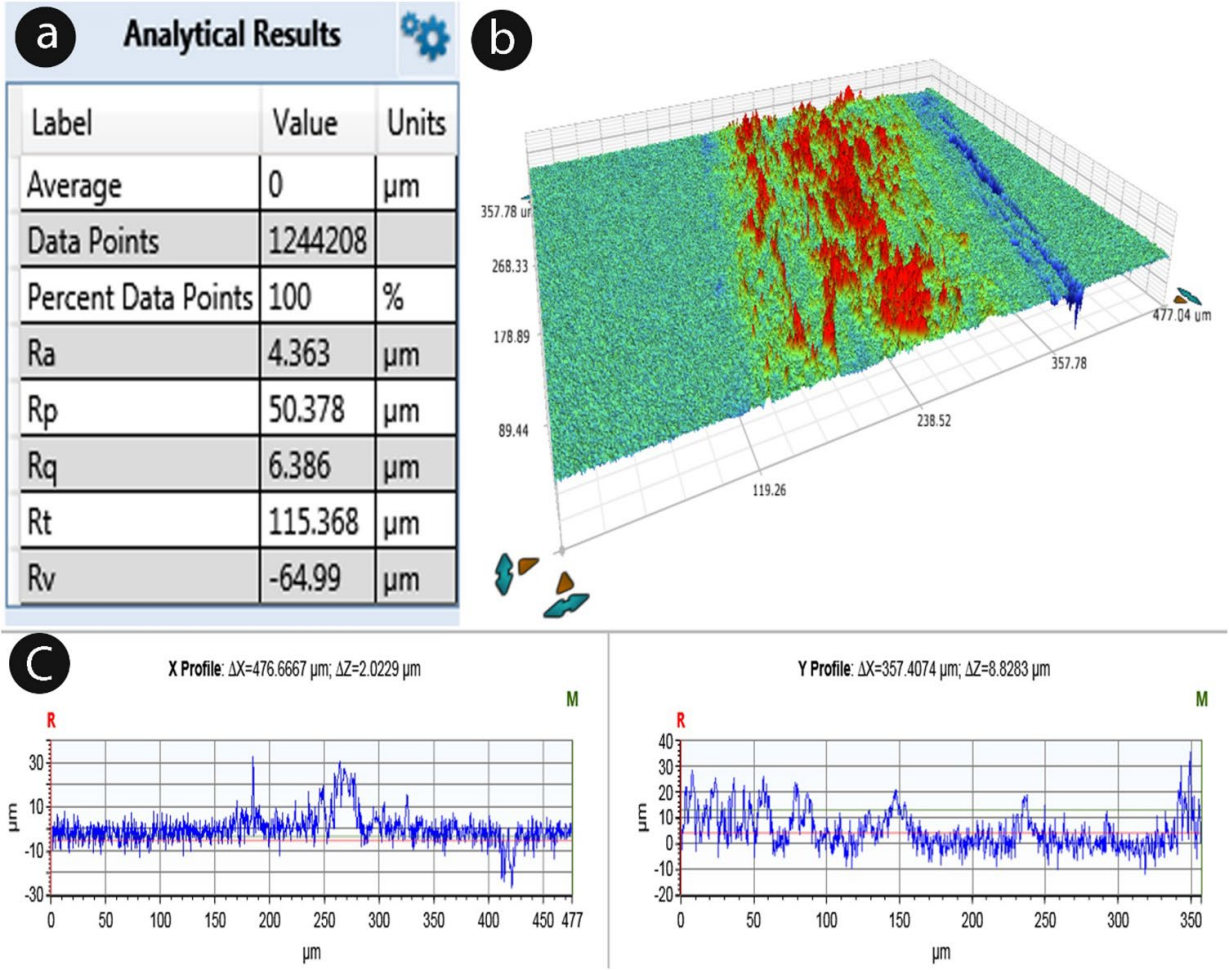
(**a**) Surface roughness analytical value (**b**) Surface roughness topographic representation (**c**) X and Y-axis profile values.

The fibrous interface's adherence to the matrix is indirectly correlated with the roughness of the surface fibrous interface's adherence to the matrix, in general, is what primarily determines the strength of fiber-reinforced materials. Moreover, higher roughness facilitates a higher deposition of contaminants. As a result, it is valuable to assess the roughness of the CPS fiber surface to predict the behaviour of interfacial adhesion. Figure 10a shows the details of the roughness parameters acquired from the 3-D measurement. Figure 10b shows the 3-D Roughness surface topology and Fig. 10c a 2-D Line model for roughness assessment. The mean average roughness (Ra) value of the CPS fiber is 4.363 µm.

## Conclusion

This study involved the extraction of new novel cellulosic fibre from the species *Cyperus platystylis*. Understanding the basic properties morphological, chemical, physical, and thermal of natural resource-based fibers is very significant to ascertaining their optimal intended uses. In the case of natural resource-based fibers greater cellulose amount and low lignin content produce greater tensile strength. The study revealed a comparatively higher percentage (%) of cellulose i.e., 66.1% in the CPS fiber, which can provide for higher tensile strength of 657 MPa and prove to be a high-quality bio-composite raw material. Tensile strength and Young's modulus both showed a favorable correlation with cellulose. Besides cellulose, hemi-cellulose and wax constituents proved to correlate positively with Young's modulus. The average mean diameter of the CPS fiber was enumerated as 23.36 μm. A thermogravimetric study revealed the thermal durability of CPS fibers up to 279 °C. The crystallinity index and crystal size of the CPS fiber are 41.12% and 2.28 nm respectively. The FTIR study and EDX analysis revealed the elemental composition with their bond characters represented in the sample. Thus, CPS fiber can be an effective contender among alternative fibers to make inroads on the share of other fibers in the worldwide natural fiber industry. The unique *Cyperus platystylis* stem fiber may be a possibility for reinforcement in bio-composites based on the examined qualities. Further, CPS fiber with nanoparticles (nanocellulose) can combine the numerous advantageous properties of cellulose coupled with the functionality of inorganic particles to yield a new intelligent material with improved properties. This fiber can thus prove to be a potent alternative to synthetic or artificial fibers and become a promising candidate for a reinforcing agent, thereby not only creating an economic value for the plant but also providing benefits to the society and environment.

## Data Availability

The datasets generated and analysed during the current study are available in the Vidyasagar University library repository (www.vidyasagar.ac.in). Kindly contact to Amal Kumar Mondal, Professor of Botany and Forestry, Vidyasagar University, Midnapore for any query related to our data. (Email: akmondal@mail.vidyasagar.ac.in).

## Abbreviations

CPS: *Cyperus platystylis* Stem
SR: Slenderness ratio
SRA: Surface Roughness Analysis
RR: Runkel ratio
CI: Crystalline Index
CS: Crystallite Size
EDX: Energy-Dispersive X-ray spectroscopy
FTIR: Fourier Transform Infrared Spectroscopy
TGA: Thermo-gravimetric Analysis
DTG: Derivative thermos-gravimetry
XRD: X-Ray Diffraction
λ: Wavelength of X-Ray
GPa: Giga Pascal
MPa: Mega Pascal
BSI: Botanical Survey of India
µm: Micrometer
mA: Milliampere
KBr: Potassium Bromide
